# Therapeutic drug monitoring of docetaxel by pharmacokinetics and pharmacogenetics: A randomized clinical trial of AUC‐guided dosing in nonsmall cell lung cancer

**DOI:** 10.1002/ctm2.354

**Published:** 2021-04-05

**Authors:** Yuxiang Ma, Xin Zhao, Xi Chen, Xinxin Huang, Qingguang Lin, Yuehao Lin, Salvatore J Salamone, Xinlan Zhou, Changzheng Wang, Weiting Liang, Hongyun Zhao, Kui Wu, Yunpeng Yang, Li Zhang

**Affiliations:** ^1^ State Key Laboratory of Oncology in South China Collaborative Innovation Center for Cancer Medicine Sun Yat‐sen University Cancer Center Guangzhou China; ^2^ BGI‐Shenzhen Shenzhen China; ^3^ Saladax Biomedical Inc. Bethlehem Pennsylvania USA

Dear Editor,

The current study is aimed to achieve precision medicine for docetaxel chemotherapy in nonsmall cell lung cancer (NSCLC) by pharmacokinetic (PK)‐ and pharmacogenetic (PG)‐based therapeutic drug monitoring (TDM). Docetaxel has a narrow therapeutic window and a huge inter‐individual variability might cause poor effects or severe toxicities.^1^ Conventional body surface area (BSA) dosing has a limited contribution in reducing PK variability.^2^ Previous research indicated that the area under the time‐concentration curve (AUC) correlated with docetaxel toxicities and efficacy, and the exposure–toxicity relationship followed the threshold mode.^3^, ^4^ Our previous study had introduced a simplified PK strategy and verified the optimal AUC range (2.5–3.7 μg·h/ml).^5^ Also, single nucleotide polymorphisms (SNPs) of critical genes are involved in taxanes biology effects, or enzymes of drug absorption, distribution, metabolism or excretion (ADME) pathways are related to docetaxel toxicities and efficacy.^6^ Herein, we conducted this randomized clinical trial to compare the safety and efficacy of PK‐guided verses BSA‐based docetaxel dosing, and post hoc analysis to explore PG biomarkers in NSCLC patients.

A total of 99 eligible patients were enrolled and randomly assigned (1:1) to receive 3‐weekly docetaxel (≤6 cycles) either with PK‐guided (arm A = 50) or 75 mg/m^2^ BSA‐guided (arm B = 49) dosing (Figure 1B). In both arms, cycle1 dose was 75 mg/m^2^ by BSA. Dose adjustments of arm B were made according to the drug label (mainly neutropenia and neuropathy). In arm A, doses adjustment from cycle 2 was obliged to follow the regulation rules (Figure 1A), based on the AUC of the previous cycle to reach the target range (2.5–3.7 μg/ml·h) and the drug label. The details of eligible criteria, assessment, and treatment are provided in Supporting Information Methods.

**FIGURE 1:**
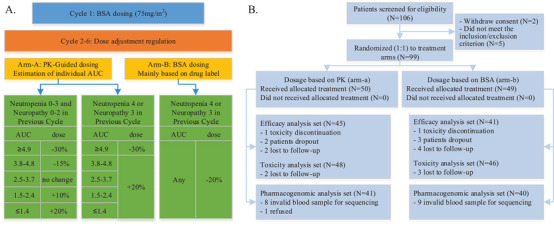
(A) Clinical trial design; PK, pharmacokinetic; AUC, area under time–concentration curve; BSA, body surface area; (B) study flowchart

A simplified PK strategy was applied, wherein docetaxel concentration of two blood samples (end‐of‐infusion −5 min and +1 h) were detected using the MyDocetaxel Assay (Saladax Biomedical Inc., Bethlehem, PA). Docetaxel AUC was calculated using nonlinear mixed‐effect modeling program.^5^ Investigators followed the regulation rules. Baseline blood samples were collected and sequenced by a custom‐designed panel covering 1042 SNP sites, including 68 genes related to biological effect and ADME pathways of docetaxel according to literature review (Table S1).

Of all 99 patients recruited in this study, basic characteristics were balanced between the two arms (Table S2). In total, patients received 175 cycles in arm A, and 137 cycles in arm B. Most patients who discontinued early were affected by toxicities in arm B (28.6% vs. 12.0%, *p *= 0.040). Details of treatment completion are shown in Table S3. The mean dose of two arms was similar in cycle 1, and separated from cycle 2 to 6 (around 61 mg/m^2^ in arm A and 72 mg/m^2^ in arm B, *p *< 0.001) (Figure 2A).

**FIGURE 2:**
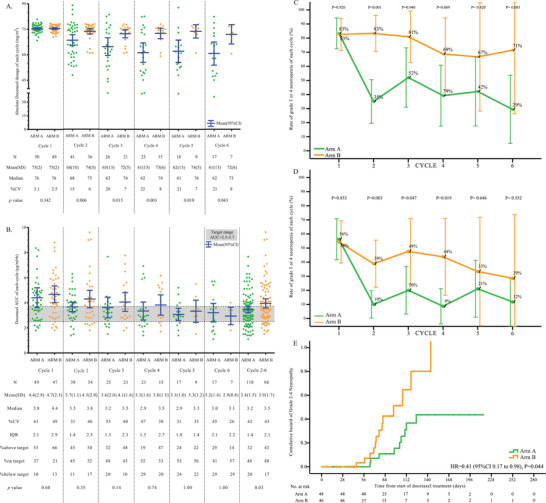
The distribution of docetaxel dose (A) and AUC (B) in each cycle of each group; N, number; SD, standard deviation; %CV, % coefficient of variation; IQR, interquartile range. (C) The grade 3–4 neutropenia rates of each cycle in arm A and B; (D) the grade 4 neutropenia rates of each cycle in arm A and B; (E) comparison of time to grade 2–4 neuropathy (cumulative hazard by treatment group), HR, hazard ratio

Previous studies described a predefined target AUC to adjust doses, and a significant decrease in neutropenia was observed after PK‐guided dosing.^7^ Similarly, our study also showed toxicities alleviation in arm A. The grade 3–4 neutropenia rates were lower in arm A from cycle 2 to 6 (29%–52%) compared to arm B (67%–83%) (Figure 2C), and maximum neutropenia severity was also lower in arm A.(G3–4 53% vs. 92%, *p *< 0.001; G4 25% vs. 50%, *p *= 0.024). Furthermore, grade 4 neutropenia rate was lower in arm A from cycle 2 to 6 (9%–21% vs. 29%–48%) (Figure 2D). The PK‐guided dose resulted in significant reduction in cumulative hazard ratio for grade 2–4 neuropathy (0.41, 95%CI 0.17–0.98, *p *= 0.044) (Figure 2E). Neuropathy rates were not significant between arm A and B. In efficacy analysis, nine PRs and seven PRs were observed in arm A and B. There were no significant differences in the objective response rate between the two groups. Median PFS in arm A and B was also not significantly different (Figure S1).

Compared to Engel's report,^7^ by enrolling a larger cohort, our study not only confirmed that patients’ PK variability was decreased by dose adjustment (both IQR and CV, Figure 2B), but also indicated that PK‐guided dosing reduced docetaxel‐related neutropenia severity and neuropathy hazard. Moreover, our study identified the value of the target range–based dose adjustment strategy of docetaxel. AUC in the PK‐guided arm entered target range earlier (cycle 2) than arm B (cycle 5), and remained within the target range. Mean AUC after PK‐guided dosing (cycle 2–6) was 3.4 μg·h/ml, well within target and significantly lower than arm B (cycle 2–6, 3.9 μg·h/ml, *p *= 0.03). The above target rate in arm A decreased from 53% in cycle 1 to 32% in cycles 2–6 (*p *= 0.012). The AUC target upper limit (3.7 μg·h/ml) introduced by previous findings was verified in the current study (Figure 3A). ROC curve revealed that AUC cutoff value by grade 3–4 neutropenia was 3.68 μg·h/ml (Figure S2).

**FIGURE 3:**
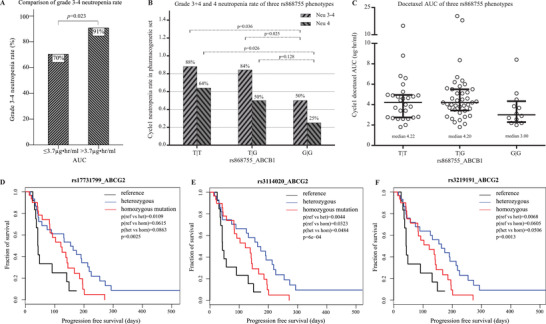
(A) The relation of docetaxel AUC in cycle 1 with grade ≥3 neutropenia rate; (B) the comparison of grade 3–4 and grade 4 neutropenia rate in patients grouped by different rs868755_ABCB1 phenotypes; (C) the comparison of AUC in patients grouped by different rs868755_ABCB1 genotypes; Kaplan–Meier curves and log‐rank comparison of progression free survival in different genotypes of three SNPs of ABCG2 [rs17731799 (D), rs3114020 (E), and rs3219191 (F)]. Reference (wild type), heterozygous and homozygous mutation

In total, 423 SNPs were observed in two‐arm combined PG correlation analysis. Ethnic population distribution of these SNP is shown in Table S4. Previous work indicated that P‐glycoprotein (ABCB1 product) is responsible for exporting xenobiotics.^8^ By regulating ABCB1 expression, some functional SNPs were associated with an increased risk of docetaxel neutropenia.^9^ We used adjusted multivariate logistic regression to find ABCB1 rs868755 significantly associated with neutropenia (*p *< 0.05) (Table S5). Further, we observed that neutropenia rate was higher in patients carrying ABCB1 rs868755 TT and TG genotypes than in those carrying GG (Figure 3B). In dominant model (TT+TG) verses recessive model (GG), the grade 3–4 neutropenia rate was 86% versus 50%, *p *= 0.018. Moreover, the AUC of docetaxel was higher in patients with dominant than recessive model (median AUC was 4.2 versus 3.0 μg·h/ml, *p *= 0.05) (Figure 3C). Relation of PG–PK–toxicities was found for the first time and ABCB1 rs868755 might be a reliable biomarker for docetaxel neutropenia.

Previous research suggested that ABCG2 pumped intracellular drug out of cells, causing a decrease in exposure and drug resistance.^10^ Our study found that ABCG2 rs17731799, rs3114020, and rs3219191 were significantly correlated with both objective response (Table S6) and PFS (Figure 3D–F). Patients who carried wild type (reference) of all three ABCG2 SNPs exhibited poor clinical efficacy and poor PFS than those who carried heterozygous or homozygous mutation. We found three ABCG2 SNPs related with docetaxel clinical efficacy, which needs further exploration.

In conclusion, the primary endpoint of the current study is met. PK‐guided dosing reduced docetaxel‐related neutropenia rate and neuropathy risk in NSCLC. PG–PK–toxicity analyses indicated that ABCB1 rs868755 dominant model is related to high docetaxel AUC and results in increasing neutropenia risk. Also, wild type of three ABCG2 SNPs is related to poor efficacy. TDM based on PK and PG analysis might improve the risk–benefit profile of docetaxel in NSCLC.

## CONFLICT OF INTEREST

X. Zhao, X. Zhou, C. Wang, and K. Wu are employees of BGI‐Shenzhen. S.J. Salamone is employee of Saladax Biomedical Inc. All remaining authors have declared no conflict of interest.

## ETHICS APPROVAL AND CONSENT TO PARTICIPATE

The study protocol was approved by independent review board of SYSUCC and written informed consent was obtained from each patient.

## DATA AVAILABILITY STATEMENT

The data of this study have been deposited into the CNGB Sequence Archive (CNSA: https://db.cngb.org/cnsa/. No. CNP0001146).

## Supporting information

Figure legends

Figure S1

Figure S2

Table S1

Table S2

Table S3

Table S4

Table S5

Table S6

Supporting Information
