# Supplementary material for: Therapeutic drug monitoring of docetaxel by pharmacokinetics and pharmacogenetics: A randomized clinical trial of AUC‐guided dosing in nonsmall cell lung cancer
Source: Clin Transl Med. 2021 Apr 5;11(4):e354. doi: 10.1002/ctm2.354 (PMC8021539; doi:10.1002/ctm2.354)
Supplement: Supplementary file 1 — Figure legends [file CTM2-11-e354-s008.docx]

**Figure legends:**

S-figure 1: A, waterfall plot of best percentage change in target lesion of both arm A and arm B, ORR, objective response rate; DCR, disease control rate; B, Progression free survival for patients in arm A and arm B.

S-figure 2: ROC curve of docetaxel AUC in cycle 1 with neutropenia severity analysis; ROC, receiver operating characteristic.
