# Supplementary figures and images for: Therapeutic drug monitoring of docetaxel by pharmacokinetics and pharmacogenetics: A randomized clinical trial of AUC‐guided dosing in nonsmall cell lung cancer
Source: Clin Transl Med. 2021 Apr 5;11(4):e354. doi: 10.1002/ctm2.354 (PMC8021539; doi:10.1002/ctm2.354)

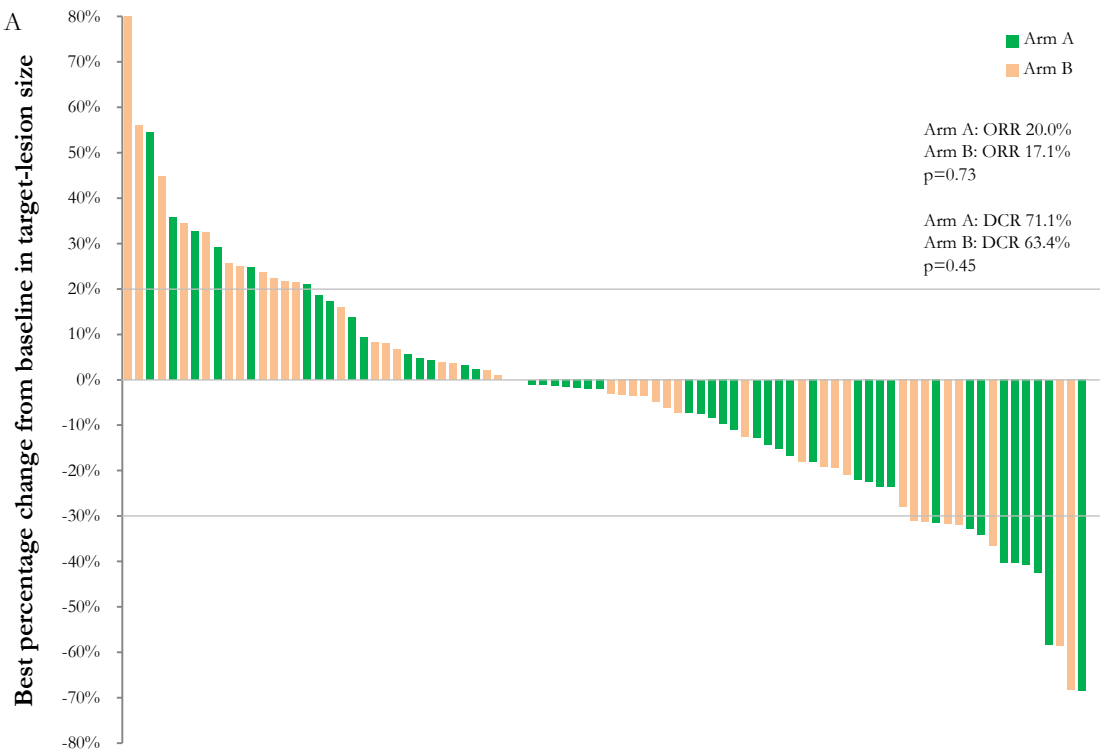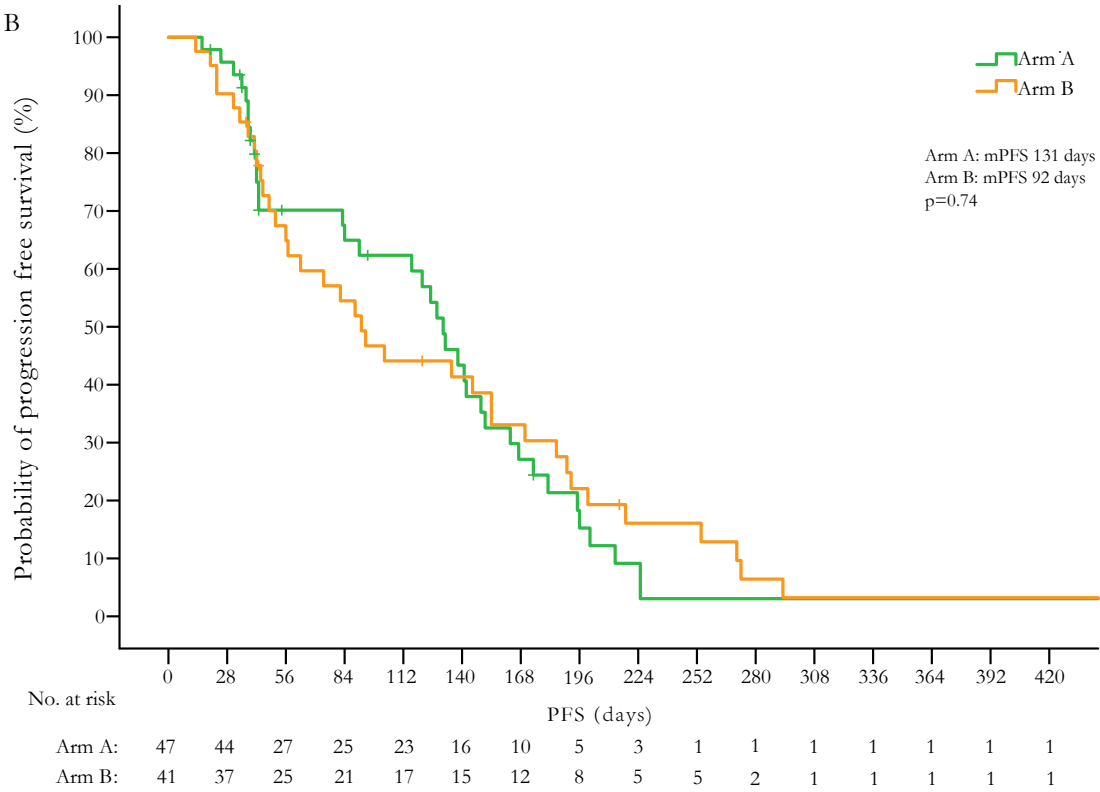

Supplement: Supplementary file 2 — Figure S1 [file CTM2-11-e354-s003.pdf]

AUC divided by neutropenia in 1<sup>st</sup> cycle (0-2 vs. 3-4)

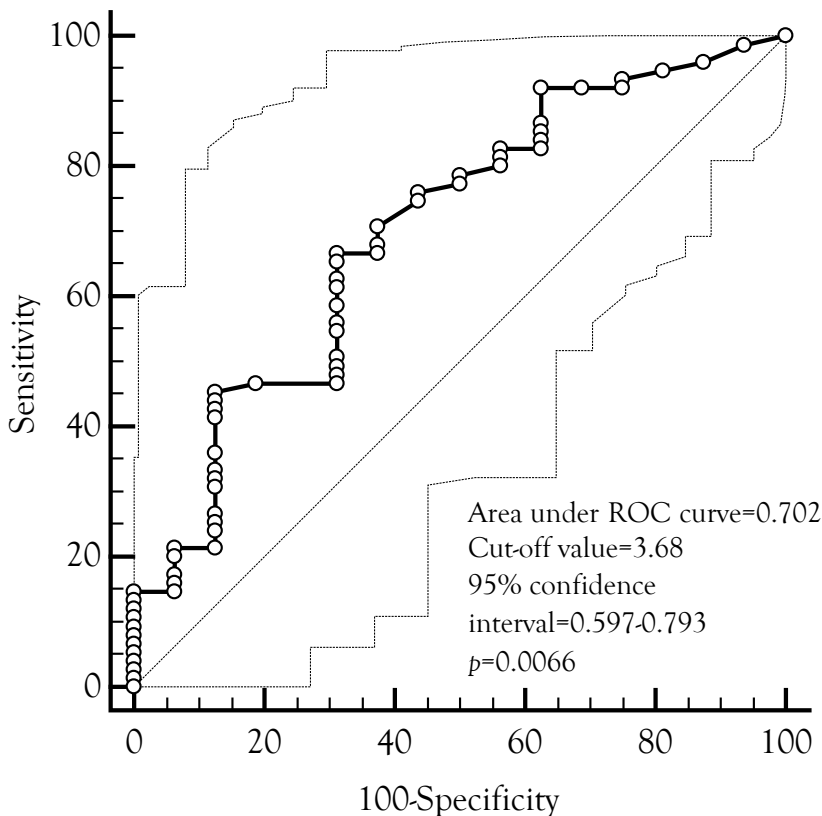

Supplement: Supplementary file 3 — Figure S2 [file CTM2-11-e354-s009.pdf]
