# Supplementary material for: Therapeutic drug monitoring of docetaxel by pharmacokinetics and pharmacogenetics: A randomized clinical trial of AUC‐guided dosing in nonsmall cell lung cancer
Source: Clin Transl Med. 2021 Apr 5;11(4):e354. doi: 10.1002/ctm2.354 (PMC8021539; doi:10.1002/ctm2.354)
Supplement: Supplementary file 5 — Table S2 [file CTM2-11-e354-s004.docx]

S-table 2: patients’ characteristics and docetaxel treatment completion of each arm.

| Characteristics | All, *N*=99 | Arm A, *N*=50  (PK-guided) | Arm B, *N*=49  (BSA) | *P* |
| --- | --- | --- | --- | --- |
| Age, years |  |  |  | 0.77 |
| Median | 54 | 55 | 53 |  |
| Range | 20-77 | 32-77 | 20-74 |  |
| Sex |  |  |  | 0.78 |
| Male | 62 | 32 | 30 |  |
| Female | 37 | 18 | 19 |  |
| BSA (m^2^) |  |  |  | 0.36 |
| Median (SD) | 1.63 (0.16) | 1.64 (0.14) | 1.61 (0.17) |  |
| Smoking status |  |  |  | 0.60 |
| Never smoked | 58 | 28 | 30 |  |
| Former/current smoker | 41 | 22 | 19 |  |
| Clinical stage |  |  |  | 0.97 |
| IIIB | 10 | 5 | 5 |  |
| IV | 89 | 45 | 44 |  |
| Distant metastasis |  |  |  | 0.94 |
| 0 | 10 | 5 | 5 |  |
| 1 | 25 | 14 | 11 |  |
| 2 | 33 | 16 | 17 |  |
| ≥3 | 31 | 15 | 16 |  |
| Performance status |  |  |  | 0.84 |
| 0 | 80 | 40 | 40 |  |
| 1 | 19 | 10 | 9 |  |
| Histology type |  |  |  | 0.68 |
| Squamous carcinoma | 14 | 7 | 7 |  |
| Adenocarcinoma | 79 | 41 | 38 |  |
| Others* | 6 | 2 | 4 |  |
| Systemic treatment lines |  |  |  | 0.63 |
| 1 | 4 | 2 | 2 |  |
| 2 | 53 | 24 | 29 |  |
| 3 | 28 | 15 | 13 |  |
| >3 | 14 | 9 | 5 |  |
| BSA, body surface area; SD, standard deviation, LELC, lymphoepithelioma like carcinoma ; LCNEC, large cell neuroendocrine carcinoma. | | | | |
