# Supplementary material for: Therapeutic drug monitoring of docetaxel by pharmacokinetics and pharmacogenetics: A randomized clinical trial of AUC‐guided dosing in nonsmall cell lung cancer
Source: Clin Transl Med. 2021 Apr 5;11(4):e354. doi: 10.1002/ctm2.354 (PMC8021539; doi:10.1002/ctm2.354)
Supplement: Supplementary file 6 — Table S3 [file CTM2-11-e354-s006.docx]

S-table 3: docetaxel treatment completion of each arm.

| Characteristics | Arm A | Arm B | P |
| --- | --- | --- | --- |
| All treatment cycles (No. %) | 175 | 137 |  |
| Cycle 1 | 50 (100) | 49 (100) | - |
| Cycle 2 | 41 (82) | 36 (73) | 0.31 |
| Cycle 3 | 26 (52) | 21 (43) | 0.36 |
| Cycle 4 | 23 (46) | 15 (31) | 0.12 |
| Cycle 5 | 18 (36) | 9 (18) | 0.05 |
| Cycle 6 | 17 (34) | 7 (14) | 0.02 |
| Early discontinued due to toxicities (No, %)* | 6 (12.0) | 14 (28.6) | 0.04 |
| Early discontinued due to loss of follow-up (No, %)Ϯ | 7 (14.0) | 7 (14.3) | 0.97 |
| Early discontinued due to patients’ dropout (No, %)Ɉ | 5 (10.0) | 3 (6.1) | 0.72 |
| Total early discontinued before PD or death (No, %) | 18 (36.0) | 24 (49.0) | 0.19 |
| Cumulative mg drug amount per patient (mean, range) | 393 (100-844) | 335 (100-850) | 0.19 |
| Cumulative mg drug amount per cycle (mean, range) | 115 (80-147) | 120 (87-145) | 0.08 |
| Median cycles per patients (No.) | 3.00 | 2.00 | 0.09 |
| Usage of G-CSF of all treatment cycles (No, %)ƪ |  |  |  |
| Cycle 1 | 28 (56) | 27 (55) | 0.93 |
| Cycle 2 | 21 (51) | 30 (83) | 0.003 |
| Cycle 3 | 17 (65) | 17 (81) | 0.24 |
| Cycle 4 | 13 (57) | 12 (80) | 0.14 |
| Cycle 5 | 11 (61) | 7 (78) | 0.67 |
| Cycle 6 | 8 (47) | 6 (86) | 0.17 |
| Delay days of subsequent cycles due to toxicity (median, range) | 3.88 (0-34) | 5.74 (0-24) | 0.16 |
| Patients with average delay > 7 days (No, %) | 7 (14.0) | 11 (22.4%) | 0.28 |
| * 2 patients discontinued due to toxicities and without radiological assessment (1 in arm-A and 1 in arm-B).  Ϯ 6 patients lost to follow-up during the treatment; 5 happened after 1^st^ cycle without any safety or efficacy data (2 in arm-A and 3 in arm-B); 1 in arm-B happened after 2^nd^ cycle without efficacy data.  Ɉ 5 patients chose early discontinuation and turned to other treatments; 5 happened prior to radiological assessment (2 in arm-A and 3 in arm-B).  ƪ Primary G-CSF prophylaxis was not permitted in the first cycle, but allowed in subsequent cycles. | | | |
