# Supplementary material for: Therapeutic drug monitoring of docetaxel by pharmacokinetics and pharmacogenetics: A randomized clinical trial of AUC‐guided dosing in nonsmall cell lung cancer
Source: Clin Transl Med. 2021 Apr 5;11(4):e354. doi: 10.1002/ctm2.354 (PMC8021539; doi:10.1002/ctm2.354)
Supplement: Supplementary file 8 — Table S5 [file CTM2-11-e354-s005.docx]

S-table 5: Genes SNPs correlated with severe neutropenia according to NCCN and MASCC guideline.

| SNP | Allele | Genetic Model | NEU 0-2 (NCCN criteria) | | | NEU 0-3 (MASCC criteria) | |
| --- | --- | --- | --- | --- | --- | --- | --- |
|  |  |  | p-value | OR | p-value | | OR |
| rs4646440  CYP3A4 | G/A | GG, GA, AA | 0.048 | 0.371 (0.139-0.992) | 0.017 | | 0.324 (0.129-0.815) |
| rs868755  ABCB1 | T/G | TT, TG, GG | 0.017 | 0.287 (0.103-0.797) | 0.026 | | 0.402 (0.180-0.895) |
| Rs4646440 G>A; rs868755 T>G; NEU, neutropenia; OR, odds ratio; NCCN, National Comprehensive Cancer Network; MASCC, Multinational Association of Supportive Care in Cancer. | | | | | | | |
