# Supplementary material for: Therapeutic drug monitoring of docetaxel by pharmacokinetics and pharmacogenetics: A randomized clinical trial of AUC‐guided dosing in nonsmall cell lung cancer
Source: Clin Transl Med. 2021 Apr 5;11(4):e354. doi: 10.1002/ctm2.354 (PMC8021539; doi:10.1002/ctm2.354)
Supplement: Supplementary file 9 — Table S6 [file CTM2-11-e354-s007.docx]

S-table 6-1: Genes SNPs correlated with ORR by logistic analysis and fisher’s exact test.

| snp | Allele | Genetic Model | Fisher | | Logistic | | |
| --- | --- | --- | --- | --- | --- | --- | --- |
|  |  |  | P-value | ODD | P-value | ODD | |
| rs17731799  ABCG2 | G/T | GG, GT, TT | 0.017 | NA | 0.012 | 0.39 (0.18-0.81) | |
|  |  | GG, GT+TT | 0.005 | 0.14(0.02-0.68) |  | NA | |
| rs212091  ABCC1 | T/C | TT, TC, CC | 0.033 | NA | 0.014 | 2.95 (1.24-6.99) | |
|  |  | TT, TC+CC | 0.026 | 3.17(1.04-10.26) |  | NA | |
| rs301927  EPHA6 | G/A | GG, GA, AA | 0.025 | NA | 0.034 | 2.62 (1.07-6.39) | |
|  |  | GG+GA, AA | 0.013 | 12.28(1.25-614.82) |  | NA | |
| rs3114018  ABCG2 |  | AA, AC+CC | 0.026 | 0.24(0.05-0.99) | 0.030 | 0.44 (0.22-0.92) | |
| rs3114020  ABCG2 | T/C | TT, TC, CC | 0.005 | NA | 0.010 | 0.38 (0.18-0.79) | |
|  |  | TT, TC+CC | 0.002 | 0.11(0.02-0.53) |  | NA | |
| rs3219191  ABCG2 | -/GTGA | TT, T\|GTGA, TGTGA\|TGTGA | 0.018 | NA | 0.028 | 0.44 (0.22-0.92) | |
|  |  | TT, T\|GTGA+TGTGA\|TGTGA | 0.005 | 0.14(0.02-0.68) |  | NA | |
| rs3787554  CYP24A1 | G/A | GG, GA, AA | 0.041 | NA | 0.016 | 2.64 (1.20-5.80) | |
|  |  | GG, GA+AA | 0.024 | 3.24(1.05-10.43) |  | NA | |
| ODD, odds ratio; NA, not applicable. | | | | | | |  |

S-table 6-2: the correlation of docetaxel SNPs and ORR.

| ORR group  No. (%) | rs17731799_ABCG2 | | | P value |
| --- | --- | --- | --- | --- |
|  | ref_GG | het_GT | alt_TT |  |
| PR/SD | 3 (27) | 21 (70) | 23 (77) | 0.011 |
| PD | 8 (73) | 9 (30) | 7 (23) |  |
|  | rs212091_ABCC1 | | |  |
|  | ref_TT | het_TC | alt_CC |  |
| PR/SD | 31 (77.5) | 15 (56) | 1(25) | 0.035 |
| PD | 9 (22.5) | 12 (44) | 3 (75) |  |
|  | rs301927_EPHA6 | | |  |
|  | ref_GG | het_GA | alt_AA |  |
| PR/SD | 19 (76) | 27 (69) | 1 (17) | 0.019 |
| PD | 6 (24) | 12 (31) | 5 (83) |  |
|  | rs3114018_ABCG2 | | |  |
|  | ref_AA | het_AC | alt_CC |  |
| PR/SD | 5 (38) | 22 (69) | 20 (77) | 0.052 |
| PD | 8 (62) | 10 (31) | 6 (23) |  |
|  | rs3114020_ABCG2 | | |  |
|  | ref_TT | het_TC | alt_CC |  |
| PR/SD | 3 (25) | 21 (75) | 22 (76) | 0.003 |
| PD | 9 (75) | 7 (25) | 7 (24) |  |
|  | rs3219191_ABCG2 | | |  |
|  | ref_T\|T | het_T\|TGTGA | alt_TGTGA\|TGTGA |  |
| PR/SD | 3 (27) | 22 (73) | 22 (73) | 0.012 |
| PD | 8 (73) | 8 (27) | 8 (27) |  |
|  | rs3787554_CYP24A1 | | |  |
|  | ref_GG | het_GA | alt_AA |  |
| PR/SD | 33 (77) | 12 (55) | 2 (33) | 0.041 |
| PD | 10 (23) | 10 (45) | 4 (67) |  |
| Rs17731799 G>T; rs212091 T>C; rs301927 G>A; rs3114018 A>C; rs3114020 T>C; rs3219191 ->GTGA; rs3787554 G>A. | | | | |
