# Supplementary material for: Therapeutic drug monitoring of docetaxel by pharmacokinetics and pharmacogenetics: A randomized clinical trial of AUC‐guided dosing in nonsmall cell lung cancer
Source: Clin Transl Med. 2021 Apr 5;11(4):e354. doi: 10.1002/ctm2.354 (PMC8021539; doi:10.1002/ctm2.354)
Supplement: Supplementary file 10 — Supporting Information [file CTM2-11-e354-s010.docx]

**Methods**

**Patients**

To be eligible, patients had to meet the following criteria:(i) 18-75 years old, (ii) pathologically confirmed with advanced (stage IIIb or IV) or recurrent NSCLC with an indication for docetaxel chemotherapy, (iii) Eastern Cooperative Oncology Group (ECOG) performance status score≤2, (iv) measurable lesion(s) per RECIST v1.1^24^, and (v) adequate organ function [neutrophil count ≥2.0 G/l, platelets≥100 G/l, total bilirubin≤1.5×upper limit of normal (ULN), AST and ALT≤2.5×ULN, and creatinine clearance≥60 ml/min]. Patient screening and treatment were conducted at Sun Yat-sen University Cancer Center (SYSUCC). Study protocols were approved by the independent review board of SYSUCC and conducted in accordance with ethical principles originating from the Declaration of Helsinki and consistent with the International Conference on Harmonisation and Good Clinical Practice guidelines. Written informed consent was obtained from each patient.

**Study design and treatment**

Eligible patients were randomly assigned at a ratio of 1:1 to receive up to 6 cycles of 3-weekly docetaxel single-agent chemotherapy, given either with standard BSA dosing (75mg/m^2^) (armB) or PK-guided dosing (armA). Docetaxel was administered by intravenous infusion after standard premedication for 1.5 h (infusion started at approximately 10-11 am). In both arms, the dose of the first cycle was 75mg/m^2^ by BSA. Regarding the dose in subsequent cycles of armB, adjustments were made according to the drug label (mainly neutropenia and neuropathy). In armA, doses adjustment was obliged to follow the regulation rules [figure 1A], were regulated based on the AUC of the previous cycle to reached the target range (2.5-3.7μg/ml·h) and drug label. Primary G-CSF prophylaxis was not permitted in the first cycle of either arm but was allowed in subsequent cycles. Patients who experienced grade 4 neutropenia or febrile neutropenia were allowed to receive therapeutic G-CSF ^25^. Docetaxel was given for up to 6 cycles until disease progression, intolerable toxicities, prior death or drop out from the trial. The planned follow-up after treatment discontinuation was conducted approximately every 2 months up to 36 months.

**Assessments**

Adverse events based on laboratory tests and physical examinations, which included a systematic assessment of neurotoxicity, prior to each cycle and each follow-up were assessed and graded by investigators per the CTCAE v4.03. Additionally, a recommended plan of complete blood count tests from peripheral blood was set at days 5, 8, 11, 14, 17 and 20 for each cycle. A planned tumor radiological assessment was performed at baseline, every 2 cycles and each follow-up by either CT or MRI. The objective response was evaluated by investigators per RECIST v1.1.

**TDM**

Two peripheral blood samples (3ml, 5min before end-of-infusion and 1h after end-of-infusion) were collected for all patients in each cycle. The plasma concentration of docetaxel was detected utilizing the MyDocetaxel™ Assay (Saladax Biomedical Inc., Bethlehem, USA)^26^. The AUC of docetaxel was calculated through simplified two-sample estimation using a nonlinear mixed-effect modeling program (version V)^17,18^. By considering the AUC of previous cycle and toxicities (neutropenia and neuropathy), investigators made the final dose adjustment decision (figure 1A).

**PG analysis**

A multiple gene panel covering 1042 SNP sites, including 68 genes related to the biological effect of taxanes and ADME pathways of docetaxel according to a literature review (s-table 1), was custom-designed. Genomic DNA (gDNA) was extracted from patients' baseline peripheral blood samples using the QIAamp DNA Blood Mini Kit (Qiagen, Germany). At least 1 µg of gDNA was fragmented and captured by a customized gene panel for library construction. The mean sequencing depth across targeted bases was 148.50×, with 91.72% of target bases above 30× coverage.

**Statistical Plan**

The primary endpoint was an improvement in the severe neutropenia rate^27^. Based on an estimated improvement in the rate of grade 3-4 neutropenia from 80% to 40%, with 80% power and two sided α of 0.05, also with 10-20% expulsion rate, totally 90-100 patients were needed for this study^14,17,27,28^. The secondary endpoints included neuropathy, the objective response rate (ORR) and progression-free survival (PFS). Patients without safety data (routine neutrophil test) will be excluded from safety analysis; without radiological assessment after baseline during treatment or follow-up will be excluded from efficacy analysis. Descriptive statistics for quantitative variables are presented as the means, standard deviations (SDs), medians, and percent coefficients of variation (%CVs), inter-quartile range (IQR) and were compared by t-tests or nonparametric tests. Categorical variables are compared with the Fisher’s exact test. PG analysis is conducted by multivariate logistic regression. A receiver operating characteristic (ROC) curve was used to analyze AUC in cycle 1. PFS was evaluated using Kaplan-Meier plots and compared between groups using the log-rank test. Multivariate logistic regression, adjusted for sex, BSA, age, and smoking status, and Fisher’s exact test were performed under the additive model, dominant model and recessive model for PG analysis. All statistical analyses were performed with R (v 3.3.2).

**SNPs selection after frequency and H-W equilibrium test**

A two-arm combined PG correlation analysis with docetaxel toxicity (neutropenia in cycle 1) and efficacy (no significant differences between 2 arms) was performed in the PG analysis set. In total, 423 SNPs were observed in81 patients; however,10 were excluded for having a frequency <10% in this cohort, 590 were excluded for having a minor allele frequency <5%, and 19 were excluded for having a p-value <0.05 (Hardy-Weinberg equilibrium). The distribution of all enrolled SNP allele frequencies in different ethnic populations is shown in s-table 4.

**SNPs selection method for neutropenia analysis**

To identify SNPs associated with neutropenia, seventy-five patients (overlap of PG and safety sets) were divided into two groups based on grades 0-2 vs. 3-4 or 0-3 vs. 4 neutropenia. We used multivariate logistic regression and adjusted for sex, age, BSA and smoking status. Two SNPs, CYP3A4 rs4646440 and ABCB1 rs868755, were significantly associated with neutropenia (p<0.05, logistic regression) (s-table 5). In particular, we observed that ABCB1 rs868755 was significantly associated with the neutropenia rate in patients grouped by genotype. The neutropenia rate was higher in patients carrying TT (wild-type, WT) and TG (heterozygous, Het) than in patients carrying GG (homozygous, Hom). The rate of grade 3-4 neutropenia was 88% (22/25) in WT patients and 84% (32/38) in Het patients (vs. 50% (6/12) in Hom patients; p=0.036 and 0.025, respectively); in the dominant model, the rate was 86% vs. 50% (WT+Het vs. Hom; p=0.018). The rate of grade 4 neutropenia was 64% (16/25) in WT patients and 50% (19/38) in Het patients (vs. 25% (3/12) in Hom patients; p=0.026 and 0.128, respectively); in the dominant model, the rate was 56% vs. 25% (p=0.052) (figure 3B).

The AUC of docetaxel was higher in patients who the carried TT and TG genotypes of ABCB1 rs868755 than in patients who carried the homozygous genotype, with a median of 4.22 and 4.20 vs. 3.00 µg•hr/ml (p=0.144 and 0.037, respectively), and a significant difference was observed in the dominant model (median 4.2 vs. 3.00 µg•hr/ml, p=0.05) (figure 3C).

**SNPs selection method for docetaxel efficacy analysis**

To evaluate the relationship between SNPs and the objective response of docetaxel, logistic regression and Fisher’s exact test were performed in 71 patients (overlap of PG and efficacy sets) which were divided into two groups according to the objective response criteria (PR+ SD vs. PD). Seven SNPs were significantly associated with the drug response (s-tables 5 and 6). Subsequently, Kaplan-Meier curves showed that ABCG2 rs17731799, rs3114020 and rs3219191 were significantly correlated with both the objective response to docetaxel and PFS (figure 5A, B, C). Compared to patients with the heterozygote and homozygote genotypes of ABCG2 rs17731799, patients with the wild-type genotype had poorer efficacy, with an ORR of 27% vs. 73%, respectively (p=0.005). The median PFS of patients with wild-type ABCG2 rs17731799 was 42.5 days, which was significantly shorter than that of patients with other mutants (Het and Hom: 132 days and 125 days, respectively). Patients who carried the wild-type genotypes of the other two SNPs also exhibited poor clinical efficacy and poor PFS (rs3114020: ORR of 25% vs. 75% and median PFS of 42 vs. 105 days in; rs3219191: ORR of 27% vs. 73% and median PFS of 42 vs. 97.5 days; p<0.05).
